# Absence of significant coronary artery disease does not eliminate smoking-related cardiovascular risk: insights from the PROMISE trial

**DOI:** 10.1016/j.ajpc.2026.101740

**Published:** 2026-07-11

**Authors:** Nóra M Kerkovits, Thomas Mayrhofer, Isabel L Langenbach, Jan M Brendel, Michelle D Kelsey, Maros Ferencik, Michael T Lu, Neha Pagidipati, Svati H Shah, Pamela S Douglas, Borek Foldyna

**Affiliations:** a Cardiovascular Imaging Research Center (CIRC), Department of Radiology, Massachusetts General Hospital, Harvard Medical School, Boston, MA, USA; b Medical Imaging Centre, Semmelweis University, Budapest, Hungary; c Center for Preventive Medicine and Digital Health, University of Heidelberg, Mannheim, Germany; d Department of Radiology, University of Tuebingen, Tuebingen, Germany; e Duke Clinical Research Institute, Duke University School of Medicine, Durham, NC, USA; f Division of Cardiology, Department of Medicine, Duke University School of Medicine, Durham, NC, USA; g Knight Cardiovascular Institute, Oregon Health & Science University, Portland, OR, USA

**Keywords:** Smoking, Inflammation, Atherosclerosis, Stress test, Coronary CT angiography, Prevention

## Abstract

**Background:**

Although the relationship between smoking and cardiovascular events is well established, it remains uncertain whether the outcomes of noninvasive testing (NIT) influence this relationship and how systemic biomarkers may aid risk stratification in patients presenting with chest pain.

**Methods:**

This study included PROMISE participants with stable chest pain and available smoking status (current, ever, or never) and NIT results (Positive NIT: ≥70% coronary stenosis on CT angiography or inducible ischemia on stress testing). Smoking was associated with systemic biomarkers (e.g., hsCRP, IL-6, MMP-9), NIT results, and major adverse cardiovascular events (MACE: cardiovascular death, myocardial infarction, unstable angina), using regression and mediation models adjusted for traditional cardiovascular risk factors (median follow-up 24.4 months).

**Results:**

Among 9100 individuals (mean age 61±8 years; 53% female), 51% were ever-smokers, including 18% current smokers. Smoking was independently associated with MACE, with positive NIT mediating this relationship (current: 16%; ever: 12%, *p* ≤ 0.006). Compared with never-smokers, smoking was associated with MACE despite negative NIT (current: aHR 2.41, 95%CI 1.49–3.89; ever: aHR 1.80, 95%CI 1.25–2.59), with the most pronounced differences seen in men vs. women and CV death/MI (sex interaction *p* = 0.047). Smoking was associated with high levels of inflammatory biomarkers, particularly MMP-9 (+51%; current vs. never-smokers), which partially mediated the smoking-MACE association (current: 25%; ever: 8%, ≤0.05).

**Conclusions:**

Negative NIT does not reduce smoking-associated MACE risk in patients with stable chest pain. Particularly, current smoking confers substantial residual cardiovascular risk, potentially mediated by MMP-9-related matrix remodeling not captured by standard testing, supporting aggressive smoking cessation irrespective of NIT results.

**Abbreviations**
ACE: Angiotensin-converting enzyme
ARB: Angiotensin-receptor blocker
ASCVD: Atherosclerotic cardiovascular disease
BMI: Body mass index
CAD: Coronary artery disease
CCTA: Coronary computed tomography angiography
CV: Cardiovascular
hsCRP: High-sensitivity C-reactive protein
IL-6: Interleukin-6
MACE: Major adverse cardiovascular events
MI: Myocardial infarction
MMP-9: Matrix metalloproteinase-9
NIT: Noninvasive testing

## 1 Introduction

Tobacco smoking is one of the leading causes of preventable death worldwide [1], accounting for roughly one-quarter of cardiovascular (CV) fatalities in the United States and 20% of deaths from coronary heart disease in the WHO European Region [2,3]. Smoking is a major CV risk factor that promotes coronary artery disease (CAD) through adverse lipid changes, thrombosis, and endothelial injury, driving atherogenesis [[4], [5], [6]]. It also induces chronic systemic inflammation, reflected by elevated inflammatory biomarkers linked to vascular remodeling pathways [[7], [8], [9], [10]]. This pro-inflammatory milieu is increasingly recognized as a key link between smoking and atherosclerotic plaque progression [7], and is associated with an increased risk of myocardial infarction (MI), stroke, and CV death [11].

Stable chest pain is a common presenting symptom among smokers. Noninvasive testing (NIT), including coronary computed tomography angiography (CCTA) and stress testing, is recommended by guidelines as a first-line assessment for patients with stable chest pain and appropriate pre-test probability, to detect obstructive CAD or evaluate inducible ischemia, and guide risk stratification [12]. However, the interplay between smoking, inflammation, significant CAD as determined by NIT, and adverse events remains incompletely understood. In particular, it is unclear whether smokers with normal NIT findings still face a higher event risk due to underlying inflammatory status or adverse plaque characteristics beyond significant CAD.

To address this knowledge gap, we utilized the PROMISE (Prospective Multicenter Imaging Study for Evaluation of Chest Pain) [13], a large multicenter study of outpatients with stable chest pain undergoing initial non-invasive cardiac testing, to assess whether cigarette smoking is related to systemic inflammation, significant CAD on NIT, and adverse cardiovascular events. We further evaluated whether inflammation serves as a significant mediator of the relationship between smoking and clinical outcomes.

## 2 Methods

### 2.1 Study design and population

This study included symptomatic outpatients without known CAD, referred to non-invasive testing and enrolled in the PROMISE trial. Briefly, the PROMISE trial was a pragmatic randomized comparative effectiveness trial that enrolled 10,003 participants across 193 North American centers between July 2010 and September 2013. PROMISE participants were randomized 1:1 to either CCTA or functional stress testing. All participants were followed for incident major adverse cardiovascular events (MACE), defined as CV death, non-fatal MI, or hospitalization for unstable angina for a minimum of 12 months [13]. As a sensitivity analysis, we evaluated a composite of hard cardiovascular events, defined as CV death or non-fatal MI. All events were adjudicated by an independent clinical events committee. For this study, participants were categorized as ever-smokers (current or former) or never-smokers based on baseline survey data. Patients with missing smoking data, those who did not undergo any diagnostic testing, or whose testing was considered non-diagnostic were excluded (Supplementary Figure 1). All patients provided written informed consent, and the main study protocol was approved by each of the local or central institutional review boards[13]. This secondary analysis was conducted with approval from the Mass General Brigham Institutional Review Board approval (IRB No 2009P002231).

### 2.2 Inflammatory biomarkers

Serum concentrations of hsCRP, IL-6, adiponectin, resistin, galectin-3, and matrix metalloproteinase-9 (MMP-9) were available in up to 3775 patients. All biomarkers were quantified using single-molecule counting technology on the Erenna platform (Singulex, Alameda, California) as described previously [14]. All assays were conducted in a centralized core laboratory, blinded to clinical data and study results.

### 2.3 Non-invasive testing

Patients underwent either CCTA or site-selected functional stress testing (exercise ECG, nuclear, or stress echocardiography) according to randomization. Diagnostic testing was carried out following professional society guidelines. Site-reported test results were classified as negative or positive NIT. CCTA was considered positive if obstructive CAD was identified (≥70% stenosis in any branch or ≥50% in the left main for primary, and ≥50% for sensitivity analysis [NIT_50_]). For patients undergoing functional testing, positive results were defined as the presence of inducible perfusion defect on nuclear stress testing, new stress-induced wall motion abnormality on echocardiogram, or ST depression on ECG, depending on the type of testing [15].

### 2.4 Statistical analysis

Continuous variables are reported as mean ± standard deviation or median with interquartile range (Q1-Q3) and compared using unpaired *t*-tests or Wilcoxon rank-sum tests. Categorical variables are presented as absolute frequencies and percentages and compared using Fisher’s exact test.

Multivariable logistic regressions were used to assess the association between smoking and NIT positivity, while Cox proportional hazards models were used to evaluate the association between smoking and MACE, including a sensitivity analysis for CV death and MI. All multivariable analyses were adjusted in stages for demographics (age, sex, and race/ethnicity), CV risk factors (hypertension, dyslipidemia, diabetes, body mass index (BMI), and family history of premature CAD). Survival analyses were also adjusted for NIT positivity.

Multivariable mediation analyses were used to evaluate whether abnormal NIT findings or selected biomarkers mediated the relationship between smoking and MACE. Biomarkers for mediation analysis were selected through an initial series of bivariable Cox regressions, including smoking and each biomarker, followed by multivariable Cox models that incorporated only those biomarkers significantly associated with MACE in the first step. Stata 18.0 (StataCorp LP) was used to perform all statistical analyses, with two-tailed p-values <0.05 considered statistically significant.

## 3 Results

### 3.1 Study population

Overall, 4659 (51%) of the 9100 included PROMISE participants were ever-smokers at baseline, with 1599 (34%) being current smokers and 3060 (66%) former smokers. Compared to never-smokers, ever-smokers were slightly younger (60±8 vs 61±8 years, *p* < 0.001), more often males (53%vs 42%, *p* < 0.001), and less often racial/ethnic minorities (20%vs 24%, *p* < 0.001). Hypertension and dyslipidemia were slightly more prevalent among never-smokers (66%vs 63%, *p* = 0.003, and 70% vs 65%, *p* < 0.001, respectively). Ever-smokers had a slightly lower BMI (30.2 ± 5.9 vs 30.7 ± 6.1 kg/m²) than never-smokers, despite leading more frequently a sedentary lifestyle (50%vs 47%, *p* < 0.001). Ever-smokers had a substantially higher median pooled cohort equation ASCVD risk score (14% [IQR: 9–23] vs 8% [IQR: 4–15], *p* < 0.001) and a two-fold higher prevalence of high-risk ASCVD category (10-year risk >20%, 32% vs 16%, *p* < 0.001). Detailed baseline characteristics are summarized in Table 1.

**Table 1:** Baseline patient characteristics stratified by smoking.

	All 9100 (100.0)	Never-smokers 4441 (48.8)	Ever-smokers 4659 (51.2)	p
Demographics				
Age, years	61±8	61±8	60±8	<0.001
Sex, women	4789 (52.6)	2592 (58.4)	2197(47.2)	<0.001
Race (racial or ethnic minority)	2000 (22.1)	1054 (23.9)	946 (20.4)	<0.001
Cardiovascular risk factors				
Hypertension	5890 (64.7)	2943 (66.3)	2947 (63.3)	0.003
Dyslipidemia	6154 (67.6)	3106 (69.9)	3048 (65.4)	<0.001
Diabetes mellitus	1935 (21.3)	966 (21.8)	969 (20.8)	0.271
Family history of premature CAD	2885 (31.8)	1378 (31.1)	1507 (32.4)	0.183
Peripheral or cerebrovascular disease	492 (5.4)	182 (4.1)	310 (6.7)	<0.001
CAD equivalent	2286 (25.1)	1096 (24.7)	1190 (25.5)	0.346
Metabolic syndrome	3436 (37.8)	1664 (37.5)	1772 (38.0)	0.589
Sedentary lifestyle	4407 (48.5)	2064 (46.5)	2343 (50.4)	<0.001
Body mass index, kg/m^2^	30.4 ± 6.0	30.7 ± 6.1	30.2 ± 5.9	<0.001
Current smoker	1599 (17.6)	-	1599 (34.3)	
Former smoker	3060 (33.6)	-	3060 (65.7)	
ASCVD risk score (PCE)	11.2 [6.1–19.6]	8.0 [4.4–14.9]	14.3 [8.8–23.2]	<0.001
Low risk (<7.5%)	2915 (32.3)	2075 (47.2)	840 (18.2)	
Intermediate risk (7.5–20%)	3921 (43.5)	1635 (37.2)	2286 (49.5)	
High risk (≥20%)	2177 (24.2)	689 (15.7)	1488 (32.3)	
Baseline medication				
β-blocker	2159 (24.8)	1063 (25.0)	1096 (24.6)	0.673
ACE-inhibitor or ARB	3811 (43.8)	1935 (45.5)	1876 (42.1)	0.001
Statin	3980 (45.7)	2006 (47.2)	1974 (44.3)	0.008
Aspirin	3886 (44.6)	1843 (43.3)	2043 (45.9)	0.019
Clinical presentation				
Primary presenting symptom				0.009
Chest pain	6620 (72.8)	3271 (73.7)	3349 (71.9)	
Dyspnea or exertion	1367 (15.0)	615 (13.9)	752 (16.2)	
Other	1107 (12.2)	553 (12.5)	554 (11.9)	
Type of angina				0.263
Typical	1042 (11.5)	495 (11.2)	547 (11.7)	
Atypical	7094 (78.0)	3454 (77.8)	3640 (78.1)	
Nonanginal pain	964 (10.6)	492 (11.1)	472 (10.1)	
Non-invasive testing				
Functional testing or CCTA				<0.001
Negative	7984 (87.7)	3954 (89.0)	4030 (86.5)	
Positive	1116 (12.3)	487 (11.0)	629 (13.5)	
Functional testing arm				0.268
Negative	4019 (87.4)	1964 (87.9)	2055 (87.4)	
Positive	582 (12.7)	270 (12.1)	312 (13.2)	
CCTA arm				<0.001
Negative	3965 (88.1)	1990 (90.17)	1975 (86.2)	
Positive	534 (11.9)	217 (9.8)	317 (13.8)	

All values are listed as mean±SD, median [IQR], or n (%). Definitions for racial minority, family history of premature CAD, CAD risk equivalent, metabolic syndrome, and sedentary lifestyle as previously published (Douglas et al., 2015). Noninvasive test strata were based on site-based diagnostic test reports (CCTA or functional stress testing). ACE: angiotensin-converting enzyme, ASCVD: atherosclerotic cardiovascular disease, ARB: angiotensin-receptor blocker, CAD: coronary artery disease, CCTA: coronary computed tomography angiography, PCE: pooled cohort equation.

### 3.2 Relationship between smoking and NIT results overall and by NIT type

Positive NIT results were more frequent among ever-smokers than never-smokers (14%vs 11%, *p* < 0.001), mainly driven by the CCTA arm (Table 1). In univariable analysis, ever-smoking (OR: 1.27, 95% CI 1.12–1.44, *p* < 0.001) and current smoking (OR: 1.25, 95% CI 1.05–1.48, *p* = 0.012) were associated with positive NIT results. After multivariable adjustment, ever-smoking remained associated with a 24% higher likelihood of positive NIT results (aOR: 1.24, 95% CI 1.09–1.42, *p* = 0.001) and current smoking with 54% higher likelihood (aOR: 1.54, 95% CI 1.28–1.85, *p* < 0.001). The association was significant in the CCTA arm (ever-smoking: aOR 1.44, 95% CI 1.19–1.74; current smoking: aOR 2.04, 95% CI 1.56–2.66; both *p* < 0.001) but not in the functional testing arm. Supplementary Table 1 provides corresponding unadjusted and adjusted regression results.

### 3.3 Association of smoking with MACE overall and in clinical subgroups

Overall, ever-smokers had nearly double the rate of adverse events compared with never-smokers (MACE: 3.0% vs 1.7%, CV death + MI: 2.0% vs 0.9%, *p* < 0.001 for both; Supplementary Table 2). In the multivariable Cox regression models, ever-smoking was associated with a nearly 60% increased risk of MACE, independent of demographic, clinical risk factors, and NIT results (aHR 1.58, 95% CI 1.19–2.09, *p* = 0.002; Model 3 in Table 2). The hazard ratio was more pronounced when analyzing the association of current smoking and MACE (aHR 1.89, 95% CI 1.30–2.74, *p* = 0.001). Even stronger associations were observed in a sensitivity analysis investigating the association of ever- and current-smoking with CV death + MI (ever-smoking: aHR 2.14, 95% CI 1.47–3.11, *p* < 0.001; current smoking: aHR 3.33, 95% CI 2.08–5.36, *p* < 0.001; Supplementary Table 3). Supplementary Figure 2 shows significant differences in MACE-free survival in ever-smokers stratified to current and former smoking status.

**Table 2:** Predictive value of smoking for MACE overall and among subgroups by NIT results.

	Univariable			Model 1			Model 2			Model 3		
	HR	95% CI	p	HR	95% CI	p	HR	95% CI	p	HR	95% CI	p
Ever-smoking												
Overall	1.78	1.35–2.35	<0.001	1.71	1.29–2.26	<0.001	1.67	1.26–2.22	<0.001	1.58	1.19–2.09	0.002
Subgroups by NIT												
Negative NIT	1.83	1.27–2.62	0.001	1.81	1.26–2.61	0.001	1.80	1.25–2.59	0.002	-		-
Positive NIT	1.46	0.94–2.26	0.091	1.38	0.88–2.14	0.156	1.29	0.83–2.01	0.259	-		-
NIT and smoking categories												
Negative NIT, never-smoker	Ref.		-	Ref.		-	Ref.		-	-		-
Negative NIT, ever-smoker	1.81	1.27–2.60	0.001	1.77	1.23–2.54	0.002	1.75	1.22–2.52	0.002	-		-
Positive NIT, never-smoker	5.70	3.61–8.99	<0.001	5.13	3.24–8.12	<0.001	5.17	3.26–8.19	<0.001	-		-
Positive NIT, ever-smoker	8.40	5.69–12.39	<0.001	7.33	4.93–10.91	<0.001	6.95	4.66–10.38	<0.001	-		-
Current smoking												
Overall	2.01	1.41–2.85	<0.001	2.19	1.52–3.15	<0.001	2.11	1.46–3.06	<0.001	1.89	1.30–2.74	0.001
Subgroups by NIT												
Negative NIT	2.10	1.34–3.30	0.001	2.37	1.48–3.81	<0.001	2.41	1.49–3.89	<0.001	-		-
Positive NIT	1.60	0.92–2.78	0.097	1.53	0.86–2.72	0.146	1.29	0.72–2.33	0.388	-		-
NIT and smoking categories												
Negative NIT, never-smoker	Ref.		-	Ref.		-	Ref.		-	-		-
Negative NIT, current smoker	2.08	1.32–3.26	0.001	2.18	1.37–3.47	0.001	2.09	1.31–3.34	0.002	-		-
Positive NIT, never-smoker	5.70	3.61–8.99	<0.001	5.19	3.27–8.23	<0.001	5.23	3.29–8.31	<0.001	-		-
Positive NIT, current smoker	9.42	5.62–15.79	<0.001	9.02	5.33–15.25	<0.001	8.52	5.03–14.45	<0.001	-		-

Model 1: age + sex + race/ethnic minority; Model 2: Model 1 + hypertension, dyslipidemia, diabetes, family history of premature CAD, BMI; Model 3: Model 2 + NIT result category. NIT: noninvasive testing.

In stratified subgroup analyses, the association between ever-smoking and adverse events tended to be higher in men compared to women with male ever-smokers having threefold increased risk of CV death + non-fatal MI compared to never-smokers (aHR 3.16, 95% CI 1.77–5.64, *p* < 0.001), whereas the association was less pronounced in women (aHR 1.48, 95% CI 0.87–2.51, *p* = 0.149), with sex being a significant interaction term (*p* = 0.047). Fig. 1 shows individual subgroup analyses for MACE and CV death + MI, respectively.

**Fig. 1:**
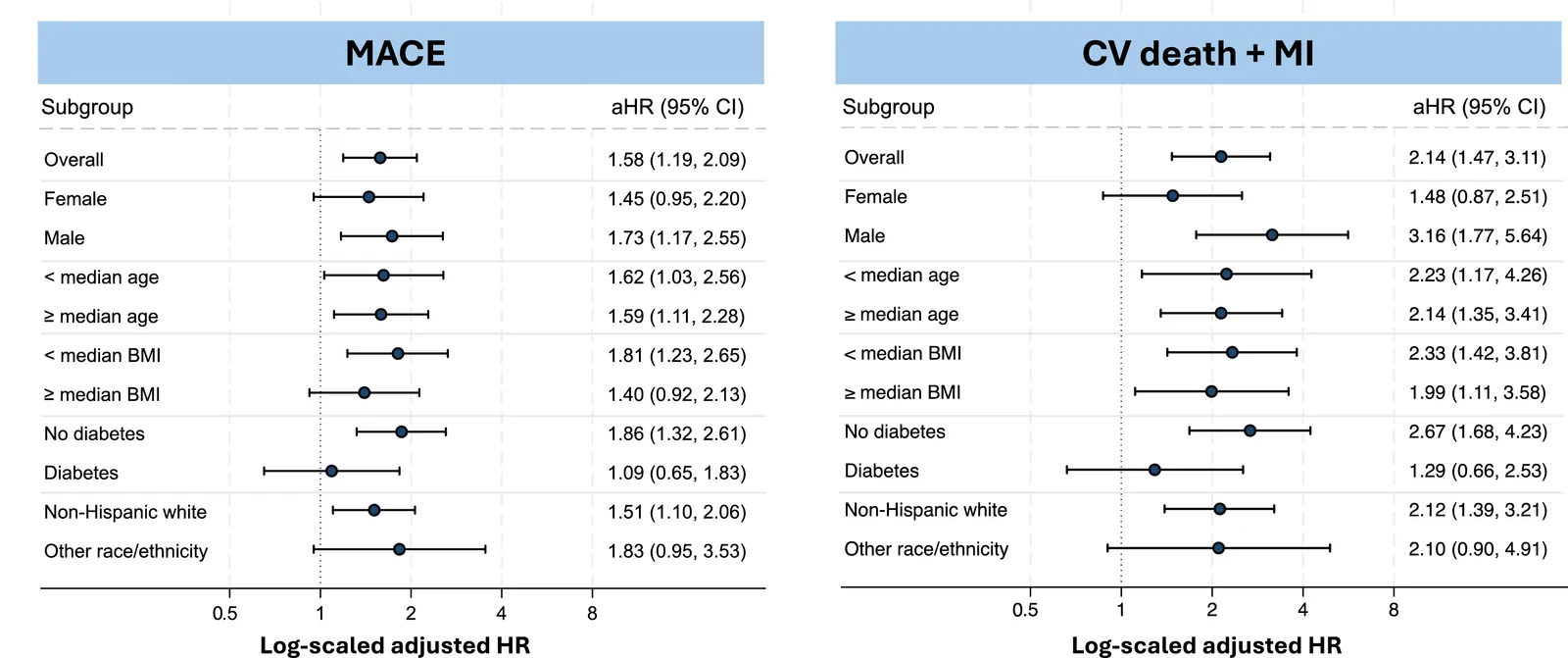
Associations of ever-smoking with MACE in patient subgroups. Multivariable Cox proportional analyses of ever-smoking in patient subgroups for the composite primary endpoint cardiovascular death, myocardial infarction, and unstable angina hospitalization. aHR: adjusted hazard ratios are controlled for age, sex, ethnic minority, hypertension, dyslipidemia, diabetes, family history of premature CAD, BMI, and significant CAD (i.e., positive NIT), always without controlling for the variable of interest. BMI: body mass index, MACE: major adverse cardiovascular events.

### 3.4 Interplay between smoking, noninvasive testing, and MACE

Overall, individuals with positive NIT experienced a 5-fold higher MACE rate and a 2.5 times higher rate of CV death + MI (*p* < 0.001 for both). Similar findings were seen across smoking categories, with 5.3x, 4.3x, and 4.3x higher MACE rates in never-smokers, ever-smokers, and current smokers, respectively. Comparable results were observed for CV death + MI, with individual event rates listed in Supplementary Table 2. The differences in MACE-free survival across NIT and smoking categories were statistically significant (unadjusted log-rank test: *p* < 0.001), as shown in Fig. 2. Notably, after adjustment for clinical covariates, ever-smokers with positive NIT had a nearly 7-fold increased risk of MACE (aHR: 6.95, 95% CI 4.66–10.38, *p* < 0.001), while current smokers had over a 8-fold higher risk of MACE (aHR: 8.52, 95% CI 5.03–14.45, *p* < 0.001) compared to never-smokers with negative NIT (Model 2, Table 2). The results were similar for secondary analyses in which positive NIT was defined as a stenosis ≥50% for the CCTA arm (Supplementary Tables 2–3). A sensitivity analysis using hard CV events (CV death and MI) revealed similar results, with a 4-fold and nearly 6-fold higher risk of CV death + MI in ever-smokers and current smokers, respectively, as detailed in Supplementary Table 4. No statistically significant interaction was observed between smoking and NIT positivity with respect to outcomes (MACE: *p* = 0.362; CV death + MI: *p* = 0.245). While there was a significant interaction between smoking and testing modality with respect to NIT positivity (*p* = 0.010), this interaction was not significant in terms of outcomes (MACE: *p* = 0.985; CV death + MI: *p* = 0.332).

**Fig. 2:**
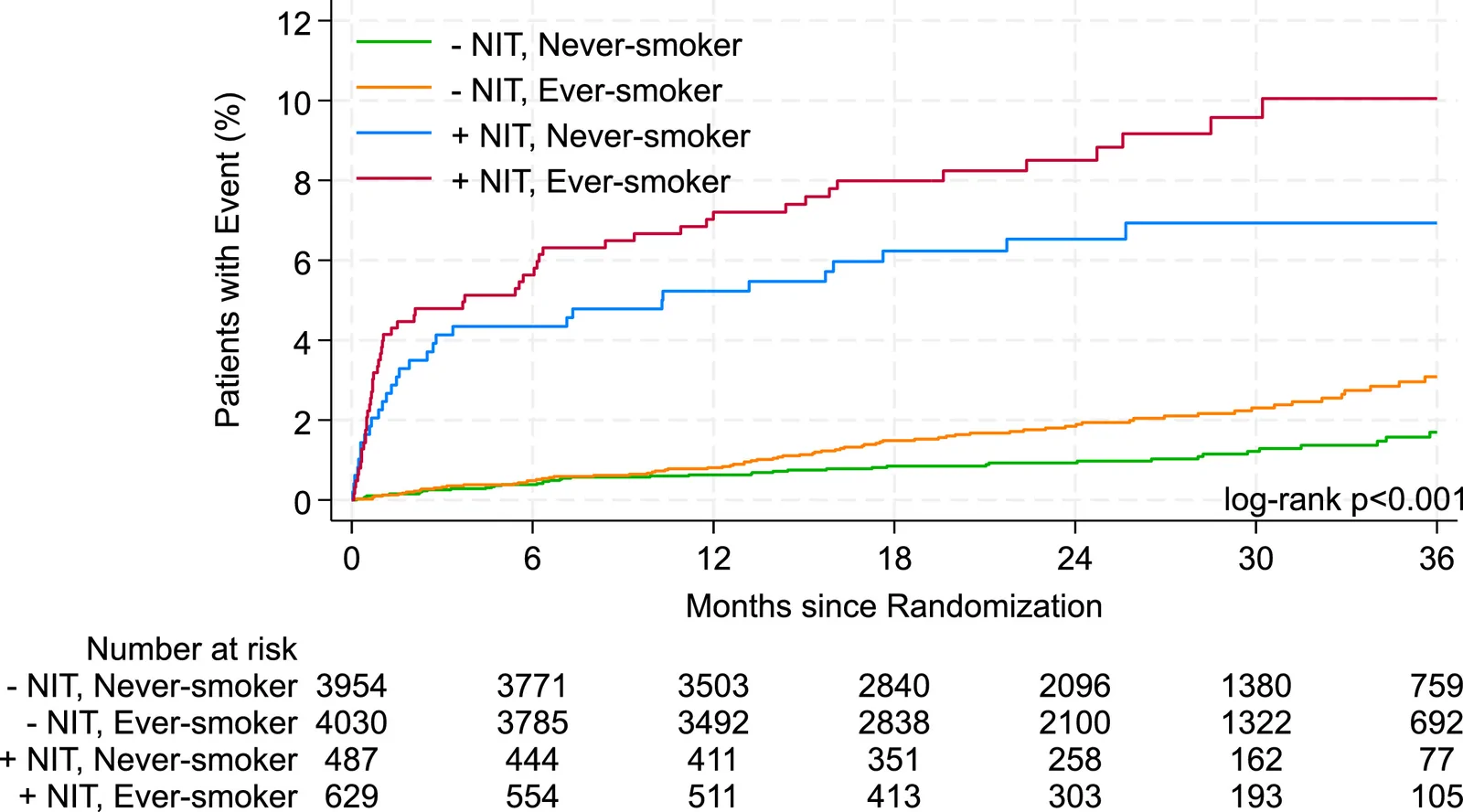
Kaplan-Meier curves by ever-smoking and noninvasive test result categories. Unadjusted Kaplan-Meier curves of MACE (cardiovascular death, myocardial infarction, and unstable angina hospitalization), stratified by smoking and noninvasive test results. NIT: non-invasive testing.

Mediation analysis revealed that 12% of the total effect of ever-smoking and 16% of current smoking on MACE was mediated by the presence of significant coronary artery disease (i.e., positive NIT results) (Fig. 3A and Supplementary Table 5).

**Fig. 3:**
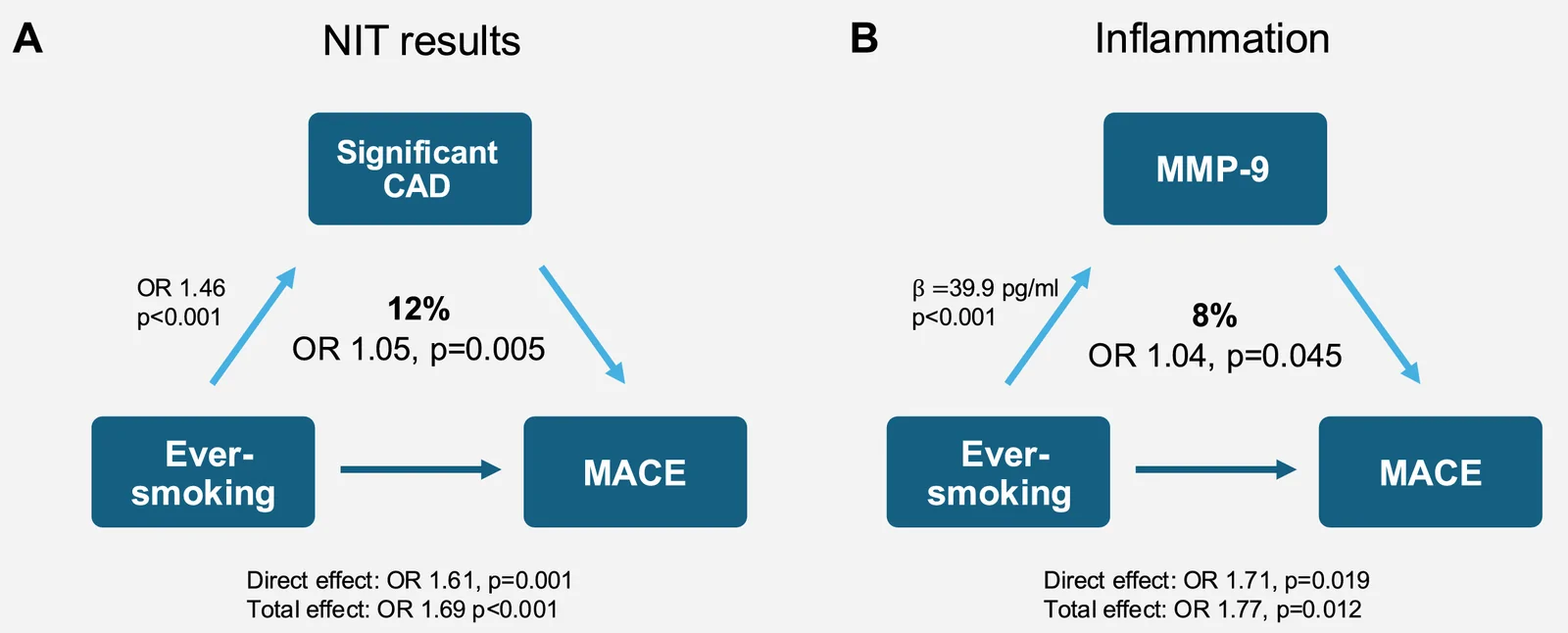
Mediators of the effect of smoking on MACE. All effects are adjusted for age, sex, hypertension, dyslipidemia, and diabetes. Sample sizes: Panel A, *n* = 9100; Panel B, *n* = 3743. Significant CAD is defined as abnormal NIT. MACE: major adverse cardiovascular events (cardiovascular death, myocardial infarction, and unstable angina hospitalization composite); CAD: coronary artery disease, NIT: noninvasive testing, MMP-9: matrix metalloproteinase-9.

### 3.5 Associations of inflammatory biomarkers with smoking, NIT results, and MACE

Systemic inflammatory biomarkers were available for a subset of PROMISE patients, ranging from 3551 to 3775 individuals. Ever-smokers had higher baseline levels of hsCRP (+9%), IL-6 (+12%), resistin (+3%), and MMP-9 (+22%) than never-smokers (*p* ≤ 0.024). In current smokers, hsCRP, IL-6, and MMP-9 were further elevated, while the protective biomarker adiponectin was significantly lower compared to never-smokers (−5%, *p* = 0.012).

Similar patterns were observed in the subgroup of patients with negative NIT results. In contrast, among those with positive NIT results, only IL-6 and MMP-9 were higher and galectin-3 was slightly lower in ever-smokers compared to never-smokers (*p* ≤ 0.037 for differences). The reduction in adiponectin among current smokers was even more pronounced in those with positive vs negative NIT results (7.5 vs 9.9 ug/ml, *p* = 0.002 for difference). Table 3 provides the individual biomarker values overall and by smoking status. Notably, hsCRP, IL-6, and MMP-9 median levels among ever-smokers with normal NIT were similar to those in never-smokers with positive NIT (*p* ≥ 0.087, Supplementary Table 6).

**Table 3:** Inflammatory biomarkers stratified by smoking history and noninvasive testing.

Median [IQR]	All	Never-smokers	Ever-smokers	p*	Current smokers	p*
Overall						
hsCRP, mg/l	2.4 [1.2–4.9]	2.3 [1.1–4.6]	2.5 [1.2–5.3]	0.008	2.7 [1.2–5.8]	0.009
IL-6, pg/ml	1.8 [1.2–2.8]	1.7 [1.1–2.6]	1.9 [1.2–2.9]	<0.001	2.0 [1.3–3.0]	<0.001
Adiponectin, ug/ml	10.3 [6.2–16.3]	10.3 [6.2–16.9]	10.3 [6.2–15.8]	0.204	9.8 [5.6–15.3]	0.006
Resistin, ng/ml	11.9 [8.9–15.6]	11.8 [8.8–15.3]	12.1 [9.2–16.0]	0.023	12.0 [9.2–16.1]	0.189
Galectin-3, ng/ml	15.0 [12.2–18.2]	15.1 [12.2–18.2]	14.9 [12.2–18.2]	0.845	14.8 [12.1–17.9]	0.448
MMP-9, ng/ml	68.8 [40.4- 115.5]	62.3 [36.8- 104.1]	75.9 [44.6–128.5]	<0.001	93.8 [57.9–159.5]	<0.001
Negative NIT						
hsCRP, mg/l	2.4 [1.2–4.8]	2.3 [1.1–4.5]	2.5 [1.2–5.3]	0.015	2.6 [1.2–5.8]	0.034
IL-6, pg/ml	1.7 [1.1–2.7]	1.6 [1.1–2.6]	1.9 [1.2–2.9]	<0.001	2.0 [1.3–2.9]	<0.001
Adiponectin, ug/ml	10.5 [6.2–16.5]	10.5 [6.2–17.0]	10.5 [6.2–16.2]	0.445	9.9 [6.1–15.4]	0.033
Resistin, ng/ml	11.9 [8.9–15.4]	11.8 [8.7–15.1]	11.9 [9.1–15.9]	0.045	11.8 [9.2–16.0]	0.196
Galectin-3, ng/ml	14.9 [12.2–18.2]	15.0 [12.1–18.1]	14.9 [12.2–18.2]	0.611	14.8 [12.2–17.9]	0.832
MMP-9, ng/ml	67.9 [40.1–114.0]	61.6 [36.5–101.9]	75.2 [44.4–127.7]	<0.001	94.2 [57.5–157.8]	<0.001
Positive NIT						
hsCRP, mg/l	2.5 [1.2–5.0]	2.6 [1.1–5.0]	2.5 [1.4–5.2]	0.349	3.3 [1.4–6.1]	0.086
IL-6, pg/ml	2.0 [1.4–3.3]	1.9 [1.3–3.1]	2.2 [1.5–3.5]	0.031	2.3 [1.7–3.5]	0.014
Adiponectin, ug/ml	9.2 [6.0–14.6]	9.8 [6.2–15.4]	8.75 [5.8–14.5]	0.169	7.5 [4.8–13.7]	0.046
Resistin, ng/ml	12.5 [9.7–16.8]	11.9 [9.6–16.8]	12.9 [9.8–16.8]	0.375	12.4 [9.5–16.3]	0.869
Galectin-3, ng/ml	15.3 [12.3–18.5]	15.6 [12.8–20.0]	14.9 [12.0–18.1]	0.037	14.7 [11.7–17.9]	0.092
MMP-9, ng/ml	73.7 [43.4–127.3]	69.5 [38.3–114.9]	78.6 [46.2–143.0]	0.031	91.5 [58.2–175.9]	<0.001

Data is presented as medians (IQR). *p-values calculated relative to never-smokers. Ever-smokers are defined as former or current smokers. Adiponectin, resistin and galectin-3 were *n* = 3728, *n* = 3719 and *n* = 3775, respectively. hsCRP: high-sensitivity C-reactive protein (*n* = 3551), IL-6: interleukin-6 (*n* = 3733), MMP-9: matrix metalloproteinase-9 (*n* = 3743), NIT: noninvasive testing.

In a multivariable Cox regression model incorporating all available inflammatory biomarkers, MMP-9 (ng/ml) was the only biomarker remaining significant as an independent variable (HR 1.002, 95% CI 1.001–1.004, *p* = 0.002). Mediation analysis, adjusted for demographics and clinical risk factors, identified MMP-9 as a significant mediator of the association between ever-smoking and MACE, accounting for 8% of the total effect through this indirect pathway (Fig. 3B). Among current smokers, the proportion mediated by MMP-9 was 25% (Supplementary Table 5).

### 3.6 Associations of current vs. former smoking with NIT results and MACE

Current smokers were substantially younger than former smokers (57±7 vs 62±8 years), and were less likely to have hypertension (57%vs 66%) or dyslipidemia (57%vs 70%); all *p* < 0.001. Although the proportion of patients with positive NIT was similar among former and current smokers (13.6%vs 13.3%, *p* = 0.821), adjusted logistic regression demonstrated that current smokers were 30% more likely to have a positive NIT than former smokers (aOR 1.30, 95% CI 1.07–1.58, *p* = 0.008). Current smokers also exhibited a less favorable biomarker profile, including approximately 40% higher MMP-9 levels. Compared with former smokers, current smokers had a 49% higher risk of MACE and an 89% higher risk of CV death or MI, independent of demographics and traditional CV risk factors. Among smokers with negative NIT results, current smokers experienced nearly two-fold higher risks of MACE and CV death+MI (Supplementary Tables 7–8).

## 4 Discussion

Clinical assessment of cardiovascular risk factors, including smoking status, and noninvasive testing for ischemia or obstructive CAD are standard first-line assessments in patients with stable chest pain. While the association between smoking and MACE is well established, this study examined how smoking relates to adverse events across NIT result strata and explored its interplay with systemic inflammation, NIT-detected significant CAD, and MACE to refine cardiovascular risk assessment and guide management strategies.

The present study yielded four main findings. First, smoking, particularly current smoking nearly doubles the risk of MACE and triples the risk of CV death and MI, independent of other traditional CV risk factors and NIT results. Second, the relative hazard of MACE associated with smoking was significant among patients with negative NIT results while not reaching significance in those with positive findings, even though smokers more frequently had abnormal NIT and higher overall event rates. Third, ever and current smoking was associated with higher systemic inflammation, reflected by higher serum levels of hsCRP, IL-6, resistin, and MMP-9, with current smokers demonstrating further increases and reduced adiponectin levels. Finally, significant CAD, indicated by positive NIT results, and MMP-9 both partially mediated the relationship between smoking and MACE.

### 4.1 Smoking, significant CAD, and MACE

Smoking was associated with MACE in this cohort with stable chest pain, consistent with prior studies involving asymptomatic populations [16,17]. A slightly higher risk for hard cardiac events (CV death + MI) was observed among men, although confidence intervals overlapped, adding to the existing inconsistency in the literature regarding sex differences in the effects of smoking. Proposed explanations for sex-related differences in smoking-associated cardiovascular risk include differences in body size, lung capacity, hormonal influences, smoking behaviors, and patterns of healthcare utilization [18,19]. However, while some studies report greater smoking-related risk in women[18, 19], others have found no differences between sexes [20,21]. Moreover, these prior observations were derived primarily from population-based cohorts rather than symptomatic patients undergoing evaluation for suspected CAD. Smokers were more likely to have significant CAD detected by positive NIT overall, an association that was primarily driven by findings in the CCTA arm. This likely reflects the ability of CCTA to detect smoking-related coronary atherosclerosis that may not necessarily manifest as inducible ischemia, potentially leading to divergent findings between testing modalities, particularly in patients with intermediate (40%–70%) stenoses [22,23]. Despite these differences, the adverse prognostic impact of smoking appeared similar across testing strategies.

Significant CAD manifesting as positive NIT mediated part of smoking’s adverse effect on MACE. However, among patients with positive NIT, the impact of smoking was more apparent when outcomes were limited to CV death or MI, indicating that these events are more sensitive to smoking-related risk.

### 4.2 Risk assessment of smokers with negative NIT

Although significant CAD detected by positive NIT partially mediated smoking’s adverse effect on MACE, the most striking finding was the markedly elevated risk among smokers with negative NIT results, especially current smokers. Smokers with negative NIT had roughly double the risk of MACE and CV death or MI, compared to never-smokers, challenging the assumption that the absence of obstructive disease or ischemia confers equal reassurance across groups.

These findings align with prior data showing that patients with normal cardiac testing retain a measurable residual risk of ∼1% per year, particularly with persistent risk behaviors such as smoking [24]. Prognostic value of myocardial perfusion imaging may also be attenuated in young smokers, supporting a risk-adapted interpretation of NIT results [25].

A negative NIT result does not exclude the presence of nonobstructive plaque, high-risk plaque features, endothelial dysfunction, or microvascular disease - all of which may be particularly relevant in smokers. In the PROMISE CCTA arm, high-risk plaque features doubled the risk of MACE among patients with nonobstructive CAD [26], and subsequent studies have reported that smoking is associated with increased odds of these plaque characteristics [27,28]. Together, these observations highlight the need for more detailed evaluation in symptomatic smokers without significant stenosis, particularly using CCTA and quantitative plaque analysis, which may further clarify how smoking influences specific plaque phenotypes, improve individualized risk stratification, and identify potential therapeutic targets (e.g. non-calcified plaque burden). Furthermore, our study underlines the importance of early smoking cessation even if NIT is negative.

### 4.3 Inflammatory biomarkers among smokers

Consistent with prior studies [8,29,30], ever-smoking was further linked to higher hsCRP, resitin, IL-6 and particularly MMP-9 (+22%). MMP-9 showed the strongest link to both smoking and MACE, mediating 8% of the overall smoking-MACE relationship. Current smokers demonstrated further increases in hsCRP, IL-6, and MMP-9, along with reduced adiponectin (−5%), in line with previous reports [8,31,32]. Importantly, former smokers had 40% lower MMP-9 levels than current smokers. The mediating effect of MMP-9 was stronger in current smokers (25%), supporting the potential involvement of MMP-9 in smoking-related vascular injury via extracellular matrix degradation, plaque destabilization, and thrombosis [[33], [34], [35]].

Given that these processes can occur in the absence of flow-limiting stenosis, our findings help explain how smokers may experience acute events even with negative NIT results. Although the link between smoking, heightened inflammation, and CV risk is well established, exploring the pathophysiology of this relationship in a large prospective cohort of symptomatic patients undergoing noninvasive evaluation for suspected CAD is novel. Stable chest pain patients with positive NIT results (reflecting underlying CV disease), regardless of smoking status, had higher inflammatory biomarker levels than those with normal test results, highlighting the connection between systemic inflammation and the presence of significant CAD [[36], [37], [38]]. Notably, ever-smokers with normal NIT showed biomarker profiles comparable to never-smokers with abnormal NIT, suggesting that smoking-related inflammation and matrix remodeling may precede overt structural disease and contribute to events even in the absence of ischemia.

Although clinical trials, such as CANTOS [39], JUPITER [40], and PROVE IT-TIMI 22 [41] demonstrate benefits of targeting inflammatory pathways, no specific MMP-9 inhibitors have been approved to date, and the clinical effects of nonspecific agents such as doxycycline remain complex [42,43]. Taken together, our findings support a potential role for MMP-9 and related vascular remodeling pathways in smoking-associated cardiovascular risk and emphasize that smoking cessation remains the most effective means to reduce inflammation-driven CV vulnerability.

## 5 Limitations

Some limitations warrant consideration. We acknowledge that the different NIT methods might have differences in specificity, sensitivity and prognostic value, which may have affected our results. Furthermore, the study had a median follow-up of just over two years and a relatively small number of MACE, limiting power for some subgroup analyses. The length of the follow-up period may also have limited our ability to capture the full long-term cardiovascular consequences of smoking, potentially resulting in an underestimation of the association between smoking and cardiovascular risk. Smoking status was self-reported and lacked quantification of tobacco exposure, including pack-years or time since cessation. Consequently, the study could not account for cumulative smoking burden or recency of exposure, introducing substantial heterogeneity within the ever-smoker category and potentially attenuating the observed associations. Prospective studies should evaluate whether smoking cessation influences outcomes in symptomatic patients with negative NIT results. Our findings should be interpreted within the context of the PROMISE population, which consisted of symptomatic patients with stable chest pain referred for noninvasive cardiovascular testing. As such, these results may not be generalizable to asymptomatic individuals undergoing screening or to broader primary prevention populations.

## 6 Conclusions

Among patients with stable chest pain, ever- and current-smoking were associated with greater systemic inflammation, more frequent abnormal NIT results, and increased cardiovascular risk. Current smoking conferred the greatest hazard, more than doubling the risk of MACE and tripling the risk of CV death or MI, independent of traditional risk factors and NIT findings. The substantially lower risk observed among former vs current smokers reinforces the importance of smoking cessation as a key preventive intervention. Notably, the excess risk was particularly evident among smokers with negative NIT results, suggesting that smoking-related inflammation and vascular injury confer cardiovascular vulnerability even before overt ischemia or obstruction develops. Thus, a negative NIT provides less reassurance, and should not be interpreted as evidence of low cardiovascular risk in symptomatic smokers. These findings support maintaining aggressive preventive risk reduction efforts in this population despite negative NIT results. Integrating biomarker and imaging data, our findings further suggest that elevated MMP-9 and NIT-detected CAD may partially mediate the smoking–MACE association, elucidating potential inflammatory and remodeling-related mechanisms that warrant further investigation as potential targets for preventive intervention.

## 7 Declaration of generative AI and AI-assisted technologies in the manuscript preparation process

During the preparation of this work, the authors used ChatGPT to assist in the creation of the central illustration. The generated output was reviewed and, where necessary, modified by the authors. The authors take full responsibility for the content of the published article.

## 8 Disclosures

Jan M. Brendel is funded by the Deutsche Forschungsgemeinschaft (DFG, German Research Foundation) #540,505,270; Maros Ferencik reports consulting fees from Cleerly, HeartFlow, Elucid, and BioMarin, advisory board of Cleerly and Elucid, and stock options Elucid; Michael T. Lu reports funding to his institution from the American Heart Association, Amgen, AstraZeneca, Ionis, Johnson & Johnson Innovation, Kowa Pharmaceuticals America, National Academy of Medicine, National Heart, Lung, and Blood Institute, and Risk Management Foundation of the Harvard Medical Institutions and serving on an advisory board for Eli Lilly, outside the submitted work; Borek Foldyna reports institutional research support from NIH/NHLBI, AstraZeneca, MedImmune, Cleerly, and MedTrace, all outside the submitted work. The other authors do not have any conflict of interest.

## Data sharing statement

PROMISE data sets are available at https://biolincc.nhlbi.nih.gov/studies/promise. There are no commercial use data restrictions, and no data restrictions based on area of research.

## Previous presentation

Part of this work was presented at the AHA 2025 Scientific Sessions. 10.1161/circ.152.suppl_3.436089.

## Central illustration

**Figure:**
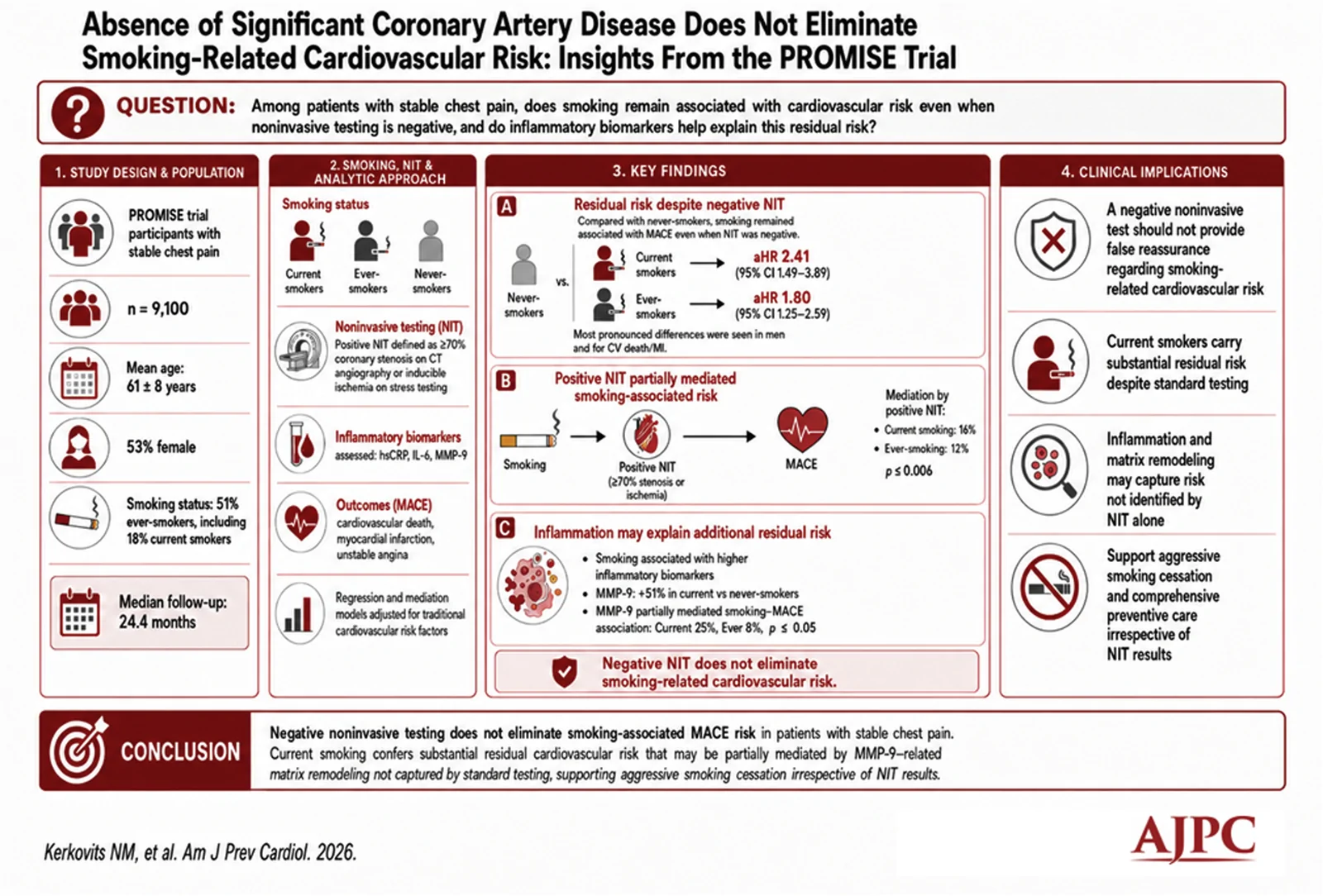
hsCRP: high-sensitivity C-reactive protein, IL-6: interleukin-6, MACE: major adverse cardiovascular events, MMP-9: matrix metalloproteinase-9, NIT: noninvasive testing.

## CRediT authorship contribution statement

**Nóra M Kerkovits:** Investigation, Writing – original draft, Visualization, Formal analysis, Data curation, Conceptualization. **Thomas Mayrhofer:** Formal analysis, Data curation. **Isabel L Langenbach:** Conceptualization. **Jan M Brendel:** Writing – review & editing. **Michelle D Kelsey:** Writing – review & editing. **Maros Ferencik:** Writing – review & editing, Conceptualization. **Michael T Lu:** Writing – review & editing. **Neha Pagidipati:** Writing – review & editing. **Svati H Shah:** Writing – review & editing. **Pamela S Douglas:** Writing – review & editing, Writing – original draft, Resources, Conceptualization. **Borek Foldyna:** Writing – review & editing, Writing – original draft, Supervision, Conceptualization.

## Declaration of competing interest

The authors declare the following financial interests/personal relationships which may be considered as potential competing interests:

Pamela S. Douglas reports financial support was provided by 10.13039/100000050National Heart Lung and Blood Institute. Borek Foldyna reports a relationship with AstraZeneca Pharmaceuticals LP, Cleerly, MedTrace, 10.13039/501100004628MedImmune that includes: funding grants. Jan M Brendel reports a relationship with 10.13039/501100001659Deutsche Forschungsgemeinschaft that includes: funding grants. Maros Ferencik reports a relationship with Cleerly Inc, 10.13039/100020588HeartFlow, 10.13039/100031637Elucid, 10.13039/100008484Biomarin that includes: consulting or advisory. Maros Ferencik reports a relationship with Elucid Bioimaging Inc. that includes: equity or stocks. Michael T. Lu reports a relationship with 10.13039/100000968American Heart Association, 10.13039/100002429Amgen, 10.13039/100004325AstraZeneca, 10.13039/100013669Ionis, Johnson & Johnson Innovation, Kowa Pharmaceuticals America, 10.13039/100009645National Academy of Medicine, NHLBI, Risk Management Foundation of the Harvard Medical Institutions that includes: funding grants. Michael T. Lu reports a relationship with 10.13039/100020536Eli Lilly that includes: consulting or advisory. If there are other authors, they declare that they have no known competing financial interests or personal relationships that could have appeared to influence the work reported in this paper.
