# Supplementary material for: Absence of significant coronary artery disease does not eliminate smoking-related cardiovascular risk: insights from the PROMISE trial
Source: Am J Prev Cardiol. 2026 Jul 11;31:101740. doi: 10.1016/j.ajpc.2026.101740 (PMC13676054; doi:10.1016/j.ajpc.2026.101740)
Supplement: Supplementary file 1 [file mmc1.docx]

**Supplementary Table 1. Association of smoking with positive noninvasive test results**

| **Positive NIT** | **Univariable** | | | **Model 1** | | | **Model 2** | | |
| --- | --- | --- | --- | --- | --- | --- | --- | --- | --- |
|  | **OR** | **95% CI** | **p** | **OR** | **95% CI** | **p** | **OR** | **95% CI** | **p** |
| **Overall** |  |  |  |  |  |  |  |  |  |
| Ever-smoking | 1.27 | 1.12-1.44 | **<0.001** | 1.22 | 1.07-1.39 | **0.002** | 1.24 | 1.09-1.42 | **0.001** |
| Current smoking | 1.25 | 1.05-1.48 | **0.012** | 1.42 | 1.18-1.70 | **<0.001** | 1.54 | 1.28-1.85 | **<0.001** |
| **CCTA arm** |  |  |  |  |  |  |  |  |  |
| Ever-smoking | 1.47 | 1.23-1.77 | **<0.001** | 1.40 | 1.16-1.69 | **<0.001** | 1.44 | 1.19-1.74 | **<0.001** |
| Current smoking | 1.57 | 1.24-2.00 | **<0.001** | 1.83 | 1.42-2.37 | **<0.001** | 2.04 | 1.56-2.66 | **<0.001** |
| **Functional testing arm** |  |  |  |  |  |  |  |  |  |
| Ever-smoking | 1.10 | 0.93-1.31 | 0.264 | 1.09 | 0.91-1.30 | 0.359 | 1.10 | 0.92-1.32 | 0.289 |
| Current smoking | 0.99 | 0.77-1.27 | 0.946 | 1.11 | 0.86-1.44 | 0.421 | 1.19 | 0.92-1.55 | 0.192 |

**Model 1:** adjusted for age, sex, and race/ethnic minority. **Model 2:** Model 1+ hypertension, dyslipidemia, diabetes, family history of premature CAD, and BMI. CCTA: coronary computed tomography angiography, NIT: non-invasive testing.

**Supplementary Table 2. Incident events overall and across noninvasive test result categories among smokers vs never-smokers**

| **n (%)** | **All** | **Never-smokers** | **Ever-smokers** | **p*** | **Current smokers** | **p*** |
| --- | --- | --- | --- | --- | --- | --- |
| **MACE** |  |  |  |  |  |  |
| **All** | 217/9,100 (2.4) | 77/4,441 (1.7) | 140/4,659 (3.0) | **<0.001** | 53/1,599 (3.3) | **<0.001** |
| **Negative NIT** | 129/7,984 (1.6) | 46/3,954 (1.2) | 83/4,030 (2.1) | **<0.001** | 32/1,386 (2.3) | **0.001** |
| **Positive NIT** | 88/1,116 (7.9) | 31/487 (6.4) | 57/629 (9.1) | 0.090 | 21/213 (9.9) | 0.094 |
| **Negative NIT_50_** | 104/7,453 (1.4) | 37/3,727 (1.0) | 67/3,726 (1.8) | **0.002** | 26/1,284 (2.0) | **0.002** |
| **Positive NIT_50_** | 113/1,647 (6.9) | 40/714 (5.6) | 73/933 (7.82) | 0.067 | 27/315 (8.6) | 0.070 |
| **CV death + MI** |  |  |  |  |  |  |
| **All** | 132/9,100 (1.5) | 40/4,441 (0.9) | 92/4,659 (2.0) | **<0.001** | 39/1,599 (2.4) | **<0.001** |
| **Negative NIT** | 99/7,984 (1.2) | 34/3,954 (0.9) | 65/4,030 (1.6) | **0.001** | 29/1,386 (2.5) | **<0.001** |
| **Positive NIT** | 33/1,116 (3.0) | 6/487 (1.2) | 27/629 (4.3) | **0.003** | 10/213 (4.7) | **0.004** |
| **Negative NIT_50_** | 84/7,453 (1.1) | 29/3,727 (0.8) | 55/3,726 (1.5) | **0.003** | 23/1,284 (1.8) | **<0.001** |
| **Positive NIT_50_** | 48/1,647 (2.9) | 11/714 (1.5) | 37/933 (4.0) | **0.003** | 16/315 (5.1) | **<0.001** |

Log-rank test was used to compare event frequencies between never-smokers and ever- or current smokers. *p-values calculated relative to never-smokers. Ever-smokers are defined as former or current smokers. Positive NIT was defined as ≥70% stenosis in any branch or ≥50% in the left main on CCTA or inducible ischaemia on functional testing. As a sensitivity analysis, event rates are also shown with positive NIT_50_ defined as a ≥50% stenosis on CCTA (and unchanged definition for functional testing). CV: cardiovascular, MACE: major adverse cardiovascular events (cardiovascular death, myocardial infarction, and unstable angina hospitalization composite), MI: myocardial infarction, NIT: non-invasive testing, NIT_50_: noninvasive testing, considered positive when ≥50% stenosis on CCTA or inducible ischaemia on functional testing.

**Supplementary Table 3. Associations of smoking with MACE overall and by NIT results, using alternative CCTA threshold of ≥50% stenosis**

|  | **Univariable** | | | **Model 1** | | | **Model 2** | | | **Model 3** | | |
| --- | --- | --- | --- | --- | --- | --- | --- | --- | --- | --- | --- | --- |
|  | **HR** | **95% CI** | **p** | **HR** | **95% CI** | **p** | **HR** | **95% CI** | **p** | **HR** | **95% CI** | **p** |
| **Ever-smoking** |  |  |  |  |  |  |  |  |  |  |  |  |
| Overall | 1.78 | 1.35-2.35 | **<0.001** | 1.71 | 1.29-2.26 | **<0.001** | 1.67 | 1.26-2.22 | **<0.001** | 1.56 | 1.18-2.07 | **0.002** |
| **Subgroups by NIT_50_** |  |  |  |  |  |  |  |  |  |  |  |  |
| Negative NIT_50_ | 1.87 | 1.25-2.80 | **0.002** | 1.89 | **1.26-2.83** | **0.002** | 1.86 | 1.24-2.79 | **0.003** | **-** |  | **-** |
| Positive NIT_50_ | 1.43 | 0.97-2.11 | 0.068 | 1.36 | 0.92-2.01 | 0.120 | 1.32 | 0.89-1.95 | 0.167 | **-** |  | **-** |
| **NIT and smoking categories** |  |  |  |  |  |  |  |  |  |  |  |  |
| Negative NIT_50_, never-smoker | Ref. |  | - | Ref. |  | - | Ref. |  | - | **-** |  | **-** |
| Negative NIT_50_, ever-smoker | 1.86 | 1.24-2.77 | **0.003** | 1.83 | 1.23-2.75 | **0.003** | 1.81 | 1.21-2.71 | 0.004 | **-** |  | **-** |
| Positive NIT_50_, never-smoker | 5.78 | 3.70-9.04 | **<0.001** | 5.19 | 3.31-8.16 | **<0.001** | 5.12 | 3.26-8.05 | **<0.001** | **-** |  | **-** |
| Positive NIT_50_, ever-smoker | 8.34 | 5.61-12.38 | **<0.001** | 7.29 | 4.86-10.92 | **<0.001** | 6.95 | 4.62-10.43 | **<0.001** | **-** |  | **-** |
| **Current smoking** |  |  |  |  |  |  |  |  |  |  |  |  |
| Overall | 2.01 | 1.41-2.85 | **<0.001** | 2.19 | 1.52-3.15 | **<0.001** | 2.11 | 1.46-3.06 | **<0.001** | 1.82 | 1.26-2.65 | **0.002** |
| **Subgroups by NIT_50_** |  |  |  |  |  |  |  |  |  |  |  |  |
| Negative NIT_50_ | 2.18 | 1.32-3.60 | **0.002** | 2.52 | 1.48-4.26 | **0.001** | 2.54 | 1.48-4.35 | **0.001** | **-** |  | **-** |
| Positive NIT_50_ | 1.56 | 0.96-2.55 | 0.73 | 1.50 | 0.90-2.50 | 0.117 | 1.39 | 0.83-2.33 | 0.209 | **-** |  | **-** |
| **NIT and smoking categories** |  |  |  |  |  |  |  |  |  |  |  |  |
| Negative NIT_50_, never-smoker | Ref. |  | - | Ref. |  | - | Ref. |  | - | **-** |  | **-** |
| Negative NIT_50_, current smoker | 2.15 | 1.30-3.55 | **0.003** | 2.25 | 1.35-3.75 | **0.002** | 2.12 | 1.27-3.59 | **0.004** | **-** |  | **-** |
| Positive NIT_50_, never-smoker | 5.78 | 3.70-9.04 | **<0.001** | 5.28 | 3.35-8.32 | **<0.001** | 5.23 | 3.31-8.27 | **<0.001** | **-** |  | **-** |
| Positive NIT_50_, current smoker | 9.08 | 5.53-14.91 | **<0.001** | 8.75 | 5.28-14.50 | **<0.001** | 8.30 | 5.00-13.78 | **<0.001** | **-** |  | **-** |

**Model 1:** age + sex + race/ethnic minority; **Model 2:** Model 1 + hypertension, dyslipidemia, diabetes, family history of premature CAD, BMI; **Model 3:** Model 2 + NIT result category.

CCTA: coronary computed tomography angiography, MACE: major adverse cardiovascular events (cardiovascular death, myocardial infarction, and unstable angina hospitalization composite), NIT_50_: noninvasive testing, considered positive when ≥50% stenosis on CCTA or inducible ischaemia on functional testing.
**Supplementary Table 4. Predictive value of smoking for the composite secondary endpoint (CV death or non-fatal MI)**

|  | **Univariable** | | | **Model 1** | | | **Model 2** | | | **Model 3** | | |
| --- | --- | --- | --- | --- | --- | --- | --- | --- | --- | --- | --- | --- |
|  | **HR** | **95% CI** | **p** | **HR** | **95% CI** | **p** | **HR** | **95% CI** | **p** | **HR** | **95% CI** | **p** |
| **Ever-smoking** |  |  |  |  |  |  |  |  |  |  |  |  |
| Overall | 2.27 | 1.56-3.29 | **<0.001** | 2.23 | 1.53-3.24 | **<0.001** | 2.18 | 1.50-3.18 | **<0.001** | 2.14 | 1.47-3.11 | **<0.001** |
| Subgroups by NIT |  |  |  |  |  |  |  |  |  |  |  |  |
| Negative NIT | 1.94 | 1.28-2.94 | **0.002** | 1.97 | 1.29-2.99 | **0.002** | 1.94 | 1.28-2.96 | **0.002** | **-** |  | **-** |
| Positive NIT | 3.58 | 1.48-8.67 | **0.005** | 3.21 | 1.32-7.82 | **0.010** | 3.02 | 1.24-7.35 | **0.015** | **-** |  | **-** |
| **NIT and smoking categories** |  |  |  |  |  |  |  |  |  |  |  |  |
| Negative NIT, never-smoker | Ref. |  | - | Ref. |  | - | Ref. |  | - | **-** |  | **-** |
| Negative NIT, ever-smoker | 1.94 | 1.28-2.93 | **0.002** | 1.93 | 1.27-2.93 | **0.002** | 1.90 | 1.25-2.88 | **0.003** | **-** |  | **-** |
| Positive NIT, never-smoker | 1.42 | 0.60- 3.38 | 0.790 | 1.22 | 0.51- 2.92 | 0.652 | 1.23 | 0.52- 2.95 | 0.639 | **-** |  | **-** |
| Positive NIT, ever-smoker | 5.17 | 3.11-8.56 | **<0.001** | 4.34 | 2.59-7.27 | **<0.001** | 4.18 | 2.50-7.01 | **<0.001** | **-** |  | **-** |
| **Current smoking** |  |  |  |  |  |  |  |  |  |  |  |  |
| Overall | 2.89 | 1.86-4.49 | **<0.001** | 3.55 | 2.23-5.66 | **<0.001** | 3.42 | 2.13-5.48 | **<0.001** | 3.33 | 2.08-5.36 | **<0.001** |
| **Subgroups by NIT** |  |  |  |  |  |  |  |  |  |  |  |  |
| Negative NIT | 2.60 | 1.58-4.27 | **<0.001** | 3.14 | 1.86-5.30 | **<0.001** | 3.22 | 1.89-5.47 | **<0.001** | **-** |  | **-** |
| Positive NIT | 3.92 | 1.42-10.78 | **0.008** | 4.82 | 1.67-13.92 | **0.004** | 3.55 | 1.18-10.66 | **0.024** | **-** |  | **-** |
| **NIT and smoking categories** |  |  |  |  |  |  |  |  |  |  |  |  |
| Negative NIT, never-smoker | Ref. |  | - | Ref. |  | - | Ref. |  | **-** | **-** |  | **-** |
| Negative NIT, ever-smoker | 2.59 | 1.58-4.25 | **<0.001** | 3.16 | 1.88-5.32 | **0.001** | 3.04 | 1.80-5.15 | **<0.001** | **-** |  | **-** |
| Positive NIT, never-smoker | 1.41 | 0.59-3.37 | 0.434 | 1.22 | 0.51-2.93 | 0.652 | 1.22 | 0.51-2.93 | 0.652 | **-** |  | **-** |
| Positive NIT, ever-smoker | 5.90 | 2.92-11.95 | **<0.001** | 6.19 | 3.01-12.69 | **<0.001** | 5.87 | 2.85-12.07 | **<0.001** | **-** |  | **-** |

**Model 1:** age + sex + race/ethnic minority. **Model 2:** Model 1 + hypertension, dyslipidemia, diabetes, family history of premature CAD, BMI. **Model 3:** Model 2 + NIT result category. NIT: non-invasive testing.

**Supplementary Table 5. Significant CAD and MMP-9 as mediators of the current smoking-MACE relationship**

| **MACE** |  | **Indirect effect** | | | **Direct effect** | | | **Total effect** | | |
| --- | --- | --- | --- | --- | --- | --- | --- | --- | --- | --- |
|  | **Proportion mediated (%)** | **OR** | **95% CI** | **p** | **OR** | **95% CI** | **p** | **OR** | **95% CI** | **p** |
| Significant CAD* | **15.6** | 1.09 | 1.03-1.16 | **0.006** | 1.97 | 1.37-2.83 | **<0.001** | 2.15 | 1.50-3.09 | **<0.001** |
| MMP-9 | **25.0** | 1.15 | 1.02-1.29 | 0.024 | 1.77 | 0.98-3.20 | 0.058 | 2.03 | 1.15-3.59 | **0.015** |

Effects are adjusted for age, sex, hypertension, dyslipidemia, and diabetes. *defined as abnormal non-invasive test. CAD: coronary artery disease, MACE: major adverse cardiovascular events (cardiovascular death, myocardial infarction, and unstable angina hospitalization composite), MMP-9: matrix metalloproteinase-9.

**Supplementary Table 6. Inflammatory biomarkers in smokers without significant CAD vs never-smokers with significant CAD (indicated by positive noninvasive testing)**

| **Overall** | **Never-smokers with positive NIT** | **Ever-smokers with negative NIT** | **p** | **Current smokers with negative NIT** | **p** |
| --- | --- | --- | --- | --- | --- |
| hsCRP, mg/l | 2.6 [1.1-5.0] | 2.5 [1.2-5.3] | 0.459 | 2.6 [1.2-5.8] | 0.357 |
| IL-6, pg/ml | 1.9 [1.3-3.1] | 1.9 [1.2-2.9] | 0.390 | 2.0 [1.3-2.9] | 0.819 |
| Adiponectin, ug/ml | 9.8 [6.2-15.4] | 10.5 [6.2-16.2] | 0.311 | 9.9 [6.1-15.4] | 0.978 |
| Resistin, ng/ml | 11.9 [9.6-16.8] | 11.9 [9.1-15.9] | 0.355 | 11.8 [9.2-16.0] | 0.380 |
| Galectin-3, ng/ml | 15.6 [12.8-20.0] | 14.9 [12.2-18.2] | **0.034** | 14.8 [12.2-17.9] | **0.021** |
| MMP-9, ng/ml | 69.5 [38.3-114.9] | 75.2 [44.4-127.7] | 0.087 | 94.2 [57.5-157.8] | **<0.001** |

*p-values calculated relative to never-smokers with positive NIT. Ever-smokers are defined as former or current smokers. hsCRP: high-sensitivity C-reactive protein, IL-6: interleukin-6, MMP-9: matrix metalloproteinase-9, NIT: noninvasive testing.

**Supplementary Table 7. Current versus former smoking: associations with positive NIT results and cardiovascular outcomes**

|  | **Univariable** | | | **Model 1** | | | **Model 2** | | | **Model 3** | | |
| --- | --- | --- | --- | --- | --- | --- | --- | --- | --- | --- | --- | --- |
| **Current vs former smoking** | **OR** | **95% CI** | **p** | **OR** | **95% CI** | **p** | **OR** | **95% CI** | **p** | **OR** | **95% CI** | **p** |
| Positive NIT | 0.98 | 0.82-1.17 | 0.795 | 1.23 | 1.02-1.49 | **0.032** | 1.30 | 1.07-1.58 | **0.008** | - |  | - |
|  | **Univariable** | | | **Model 1** | | | **Model 2** | | | **Model 3** | | |
| **Current vs former smoking** | **HR** | **95% CI** | **p** | **HR** | **95% CI** | **p** | **HR** | **95% CI** | **p** | **HR** | **95% CI** | **p** |
| **MACE** |  |  |  |  |  |  |  |  |  |  |  |  |
| Overall | 1.21 | 0.86-1.70 | 0.277 | 1.62 | 1.13-2.34 | **0.009** | 1.57 | 1.08-2.28 | **0.017** | 1.49 | 1.03-2.15 | **0.036** |
| **Subgroups by NIT** |  |  |  |  |  |  |  |  |  |  |  |  |
| Negative NIT | 1.25 | 0.80-1.94 | 0.325 | 1.77 | 1.10-2.85 | **0.019** | 1.75 | 1.08-2.85 | **0.024** | **-** |  | **-** |
| Positive NIT | 1.17 | 0.68-2.01 | 0.566 | 1.26 | 0.72-2.21 | 0.426 | 1.22 | 0.68-2.16 | 0.505 | **-** |  | **-** |
| **CV death + MI** |  |  |  |  |  |  |  |  |  |  |  |  |
| Overall | 1.47 | 0.97-2.23 | 0.066 | 2.12 | 1.36-3.30 | **0.001** | 1.95 | 1.23-3.06 | **0.004** | 1.89 | 1.20-2.98 | **0.006** |
| **Subgroups by NIT** |  |  |  |  |  |  |  |  |  |  |  |  |
| Negative NIT | 1.61 | 0.99-2.63 | 0.056 | 2.23 | 1.32-3.78 | **0.003** | 2.16 | 1.26-3.71 | **0.005** | **-** |  | **-** |
| Positive NIT | 1.20 | 0.55-2.61 | 0.653 | 1.65 | 0.72-3.78 | 0.235 | 1.41 | 0.60-3.30 | 0.433 | **-** |  | **-** |

**Model 1:** age + sex + race/ethnic minority; **Model 2:** Model 1 + hypertension, dyslipidemia, diabetes, family history of premature CAD, BMI; **Model 3:** Model 2 + NIT result category. CV: cardiovascular, MACE: major adverse cardiovascular events (cardiovascular death, myocardial infarction, and unstable angina hospitalization composite), MI: myocardial infarction, NIT: noninvasive testing.

**Supplementary Table 8. Inflammatory biomarkers stratified by current or former smoking**

| **Median [IQR]** | **Former smokers** | **Current smokers** | **p** |
| --- | --- | --- | --- |
| **Overall** |  |  |  |
| hsCRP, mg/l | 2.5 [1.3–5.1] | 2.7 [1.2–5.8] | 0.302 |
| IL-6, pg/ml | 1.8 [1.2–2.9] | 2.0 [1.3–3.0] | **0.007** |
| Adiponectin, ug/ml | 10.6 [6.3–16.3] | 9.8 [5.6–15.3] | **0.007** |
| Resistin, ng/ml | 12.1 [9.2–16.0] | 12.0 [9.2–16.1] | 0.653 |
| Galectin-3, ng/ml | 15.0 [12.3–18.4] | 14.8 [12.1–17.9] | 0.389 |
| MMP-9, ng/ml | 67.07 [39.58–110.46] | 93.8 [57.9–159.5] | **<0.001** |

hsCRP: high-sensitivity C-reactive protein, IL-6: interleukin-6, MMP-9: matrix metalloproteinase-9.

**Supplementary Figure 1. Consort diagram**

CAC: coronary artery calcium, CCTA: computed tomography angiography, PROMISE: Prospective Multicenter Imaging Study for Evaluation of Chest Pain. Functional testing: exercise ECG, nuclear, or stress echocardiography.

**Supplementary Figure 2. Kaplan-Meier curves of composite endpoints, stratified by never, current and former smoking**

**MACE**

MACE: major adverse cardiovascular events

**CV death and non-fatal MI**

CV: cardiovascular, MI: myocardial infarction.
